# Production and extraction of sugars from switchgrass hydrolyzed in ionic liquids

**DOI:** 10.1186/1754-6834-6-39

**Published:** 2013-03-20

**Authors:** Ning Sun, Hanbin Liu, Noppadon Sathitsuksanoh, Vitalie Stavila, Manali Sawant, Anaise Bonito, Kim Tran, Anthe George, Kenneth L Sale, Seema Singh, Blake A Simmons, Bradley M Holmes

**Affiliations:** 1 Deconstruction Division, Joint BioEnergy Institute, Lawrence Berkeley National Laboratory, Berkeley, CA, USA; 2 Biological and Materials Sciences Center, Sandia National Laboratories, Livermore, CA, USA; 3 Hydrogen and Combustion Technologies Department, Sandia National Laboratories, Livermore, CA, USA

**Keywords:** Sugar extraction, Ionic liquids, Acidolysis, Aqueous biphasic system

## Abstract

**Background:**

The use of Ionic liquids (ILs) as biomass solvents is considered to be an attractive alternative for the pretreatment of lignocellulosic biomass. Acid catalysts have been used previously to hydrolyze polysaccharides into fermentable sugars during IL pretreatment. This could potentially provide a means of liberating fermentable sugars from biomass without the use of costly enzymes. However, the separation of the sugars from the aqueous IL and recovery of IL is challenging and imperative to make this process viable.

**Results:**

Aqueous alkaline solutions are used to induce the formation of a biphasic system to recover sugars produced from the acid catalyzed hydrolysis of switchgrass in imidazolium-based ILs. The amount of sugar produced from this process was proportional to the extent of biomass solubilized. Pretreatment at high temperatures (e.g., 160°C, 1.5 h) was more effective in producing glucose. Sugar extraction into the alkali phase was dependent on both the amount of sugar produced by acidolysis and the alkali concentration in the aqueous extractant phase. Maximum yields of 53% glucose and 88% xylose are recovered in the alkali phase, based on the amounts present in the initial biomass. The partition coefficients of glucose and xylose between the IL and alkali phases can be accurately predicted using molecular dynamics simulations.

**Conclusions:**

This biphasic system may enable the facile recycling of IL and rapid recovery of the sugars, and provides an alternative route to the production of monomeric sugars from biomass that eliminates the need for enzymatic saccharification and also reduces the amount of water required.

## Background

Lignocellulosic biomass is a renewable resource that may be converted to fuels and/or chemicals [1,2]. Recent research and development efforts have examined a two step bioconversion process that involves: 1) liberation of fermentable sugars from lignocellulose and, 2) conversion of sugars into fuels and/or chemicals by fermentation [3,4]. The potential of lignocellulosic biomass to serve as a renewable feedstock has not been realized, primarily due to the historical shift towards petroleum-based feedstocks in the 1920s and the difficulty in depolymerization of lignocellulose into its component monomeric sugars [5].

The use of ionic liquids (ILs) as biomass solvents is an attractive alternative for the pretreatment of lignocellulosic biomass [6]. It has been shown that pretreatment with imidazolium-based ILs containing anions such as chloride [7], acetate [8] and alkyl phosphate [9], can greatly accelerate the subsequent enzymatic hydrolysis of biomass. Current approaches that use neat IL as the pretreatment solvent and water as antisolvent to precipitate carbohydrate-rich material require significant amounts of water to extract residual IL from the precipitated cellulose and undissolved biomass, and also require an effective means to recover and recycle the IL [8,10]. These requirements pose significant economic and sustainability challenges to the deployment of the IL pretreatment technology [11].

Another approach to sugar production using ILs is to use acid catalysts to produce sugars and other compounds *in situ* through the hydrolysis of polysaccharides dissolved in imidazolium chloride [12-15]. Li *et al.* reported biomass hydrolysis in ILs with different mineral acids as catalysts and achieved a maximum 81% liberation of the total reducing sugars initially present in the biomass with 1-n-butyl-3-methylimidazolium chloride ([C_4_mim]Cl) and hydrochloric acid [12]. The use of Brønsted acid ILs, which act as both the solvent and catalyst, to dissolve and hydrolyze cellulose has also been reported [16]. This could potentially provide a means of liberating fermentable sugars from biomass without employing enzymatic saccharification [11,17]. Separation of the sugars from the aqueous IL and recovery of the IL after acid hydrolysis are challenging and must be addressed in order to make this process economically viable.

Rogers *et al.* reported that certain hydrophilic ILs could form an aqueous biphasic system (ABS) in the presence of concentrated kosmotropic salts [18]. Subsequently, significant progress has been made that demonstrates the efficacy of this approach for separation of biomolecules, small organic molecules, biochemicals, and radiological isotopes [19-23]. It has been reported that an ABS can be formed with addition of an appropriate amount of K_3_PO_4_, K_2_HPO_4_, K_2_CO_3_, KOH, NaOH, or Na_2_HPO_4_ to an aqueous solution containing [C_4_mim]Cl [19,24]. When added to certain aqueous IL solutions, kosmotropic anions stabilize water-water interactions, resulting in more energy being required for cavity formation around the bulky organic [C_4_mim]^+^ cation. At a certain concentration of kosmotropic salts, an aqueous phase containing chaotropic IL can phase separate with the salt phase [23]. We describe a process that uses the phase separation behavior of imidazolium ILs/alkali/water solutions in tandem with acid catalyzed hydrolysis to extract the sugars liberated from biomass from the aqueous IL solutions. This approach offers the potential of reducing costs of sugar production from lignocellulose by eliminating the need for enzymes and decreasing the water consumption requirements of more traditional IL pretreatment approaches.

## Results and discussion

### Alkali extraction using sugar standards

**Figure 1:**
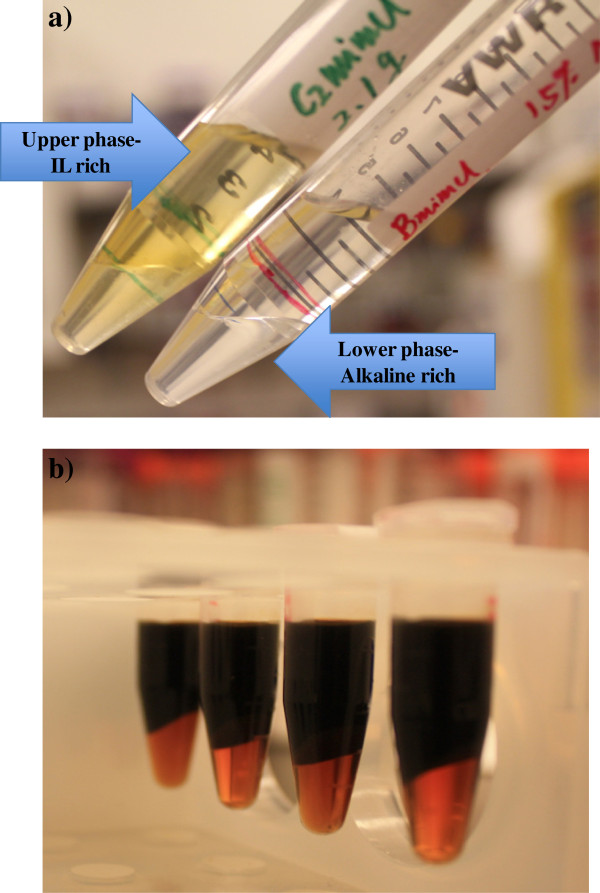
**Phase separation with addition of 15% NaOH to the ILs: a) no biomass, left-[C**_**2**_**mim]Cl, right-[C**_**4**_**mim]Cl; b) after acidolysis of biomass.**

As shown in Figure 1, clear phase separation was obtained with addition of 2 mL of 15% NaOH to 2 g IL ([C_2_mim]Cl or [C_4_mim]Cl). In the absence of glucose (or switchgrass), 10 wt% NaOH phase separated with both [C_2_mim]Cl or [C_4_mim]Cl. However, upon addition of switchgrass, no clear phase separation was observed with a final NaOH concentration of 10 wt%. Therefore, 15 wt% NaOH was used for processing switchgrass.

**Figure 2:**
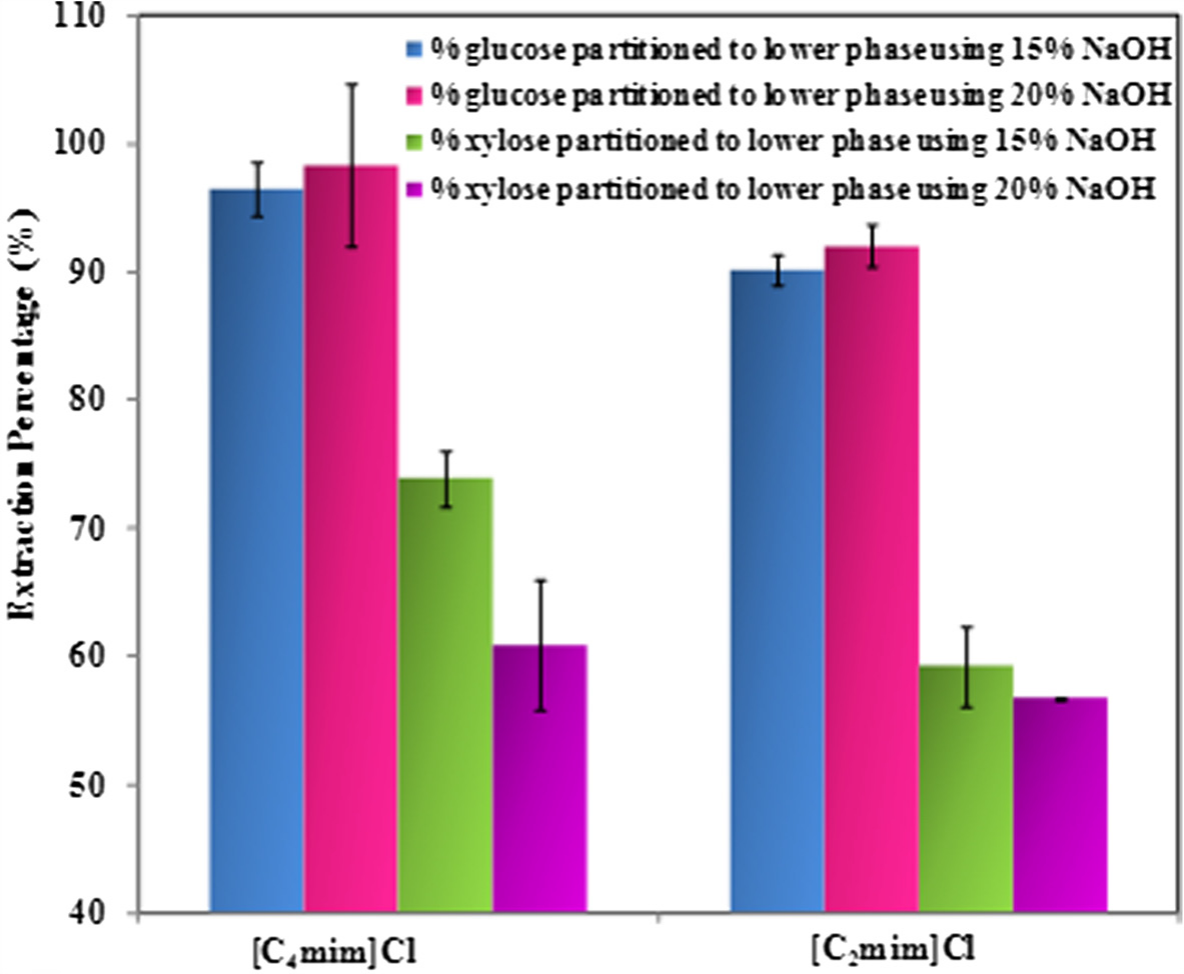
Percentage of glucose and xylose partitioned to the lower alkaline rich phase using two different NaOH concentrations.

In order to determine the partition coefficients of sugars in water-IL solutions, mixtures of glucose (0.033 g) and xylose (0.021 g) were added to [C_2_mim]Cl or [C_4_mim]Cl aqueous solutions (ca 43% H_2_O, sugar concentrations are equivalent to 5 wt% switchgrass loading). To mimic acid hydrolysis conditions, 70 uL of 4 M HCl was added to the mixture. After dissolution, a calculated amount of concentrated NaOH (50 wt%) was added. The final concentration of NaOH in the system was either 15 wt% or 20 wt%, and the sugar analysis results are plotted in Figure 2. The reported results (Extraction%) are the percentage of the sugars in NaOH phase to the sugars in IL aqueous solution before phase separation calculated using Eq. 1:

(1) $$\mathrm{Extraction}\;\%=\frac{C_{\mathrm{low}}\times V_{\mathrm{low}}}{C_{\sup}\times V_{\sup}}\times 100\%$$

where *C*_*low*_ is the sugar concentration of the bottom (alkaline rich) phase, *V*_*low*_ is the volume of the bottom phase, *C*_*sup*_ is the sugar concentration of the supernatant before the addition of NaOH, and *V*_*sup*_ is the volume of the supernatant used for phase separation (1 mL).

Sugar concentrations before addition of NaOH correspond to 90–100% of the sugars added to the aqueous [C_2_mim]Cl or [C_4_mim]Cl solution, indicating minimal degradation of sugars under the conditions used for acidolysis. It was observed that more glucose is extracted to the bottom phase in comparison to xylose. For the upper IL rich phase, less than 1% glucose or xylose can be detected. The chromatograms of the upper and lower phases are shown in Additional file 1: Figure S1. It should be noted that due to the significantly different sugar concentrations present, the lower phase aliquot was diluted 3000× in order to be quantified by High Performance Anion Exchange Chromatography (HPAEC); however, the upper phase was only diluted 5×. The system using [C_4_mim]Cl was found to be more efficient, with better extractions for both glucose and xylose ([C_4_mim]Cl with 15% NaOH: 96.5% for glucose, 73.9% for xylose vs. [C_2_mim]Cl with 15% NaOH: 90.1% for glucose, 59.2% for xylose). With higher concentrations of NaOH, the amount of glucose partitioned to the lower phase is slightly higher ([C_4_mim]Cl: 96.5% with 15% vs. 98.3% with 20%; [C_2_mim]Cl: 90.1% with 15% vs. 92.0% with 20%), while the amount of xylose in the bottom phase decreased ([C_4_mim]Cl: 73.9% with 15% vs. 60.9% with 20%; [C_2_mim]Cl: 59.2% with 15% vs. 56.7% with 20%). We hypothesized that this was due to the degradation of the xylose in strongly basic conditions. Based on the results obtained with sugar standards, [C_4_mim]Cl/15% NaOH system was selected as the system for subsequent experiments with switchgrass.

Partitioning of glucose into the alkaline phase is driven by favorable interactions with the NaOH/water system as compared to the IL/water system. To evaluate the ability to accurately predict this partitioning based on the energetics of the interactions glucose has with the two solution environments, 10 ns molecular dynamics (MD) simulations were run from which the partition coefficient for glucose in each of the two phases was calculated according to Eqs. 2 and 3 using the last 8 ns of the simulation (Table 1). The partition coefficients are calculated from the differences in interaction energies of glucose with different solvent systems:

(2) $$\log P=\frac{\mathrm{\Delta \Delta G}}{2.3\times R\times T}$$

where the difference in interaction energy is calculated as:

(3) $$\begin{array}{l} \mathrm{\Delta \Delta G}\approx \mathrm{\Delta \Delta E}=\left(E_{\mathrm{sugar},\mathrm{NaOH}\;\mathrm{phase}}-E_{\mathrm{sugar}}-E_{\mathrm{NaOH}\;\mathrm{phase}}\right)\\ \;-\left(E_{\mathrm{sugar},\mathrm{IL}\;\mathrm{phase}}-E_{\mathrm{sugar}}-E_{\mathrm{IL}\;\mathrm{phase}}\right)\\ \;=\left(E_{\mathrm{sugar},\mathrm{NaOH}\;\mathrm{phase}}-E_{\mathrm{NaOH}\;\mathrm{phase}}\right)\\ \;-\left(E_{\mathrm{sugar},\mathrm{IL}\;\mathrm{phase}}-E_{\mathrm{IL}\;\mathrm{phase}}\right) \end{array}$$

**Table 1:** The total potential energies of the simulated systems

	**[C**_**2**_**mim]Cl (kcal/mol)**	**NaOH (kcal/mol)**
Sugar + system	−3998.02	−22477.84
Solvent only	−4500.37	−22948.28
ΔE	502.36	470.44
ΔΔE (per glucose)	−3.99	

The calculated interaction energy of glucose with the NaOH/water systems is more preferred (lower interaction energy) than the interaction energy of glucose in the IL/water system, indicating that glucose tends to partition into the alkaline phase. The more preferred interactions between glucose and NaOH/water solutions also indicates that the hydroxyl group forms stronger hydrogen bonds with the anionic OH^-^ group due to the stronger charge distribution around the OH^-^ anions. The calculated partition coefficient is 2.91 and agrees well with the experimental result of 3.18. These results suggest that MD simulations can be used to understand the partitioning of monomeric sugars into various IL/water and NaOH/water systems and to predict partition coefficients of sugars into these systems to assist in the choice of the IL.

### Biomass pretreatment and acidolysis

**Figure 3:**
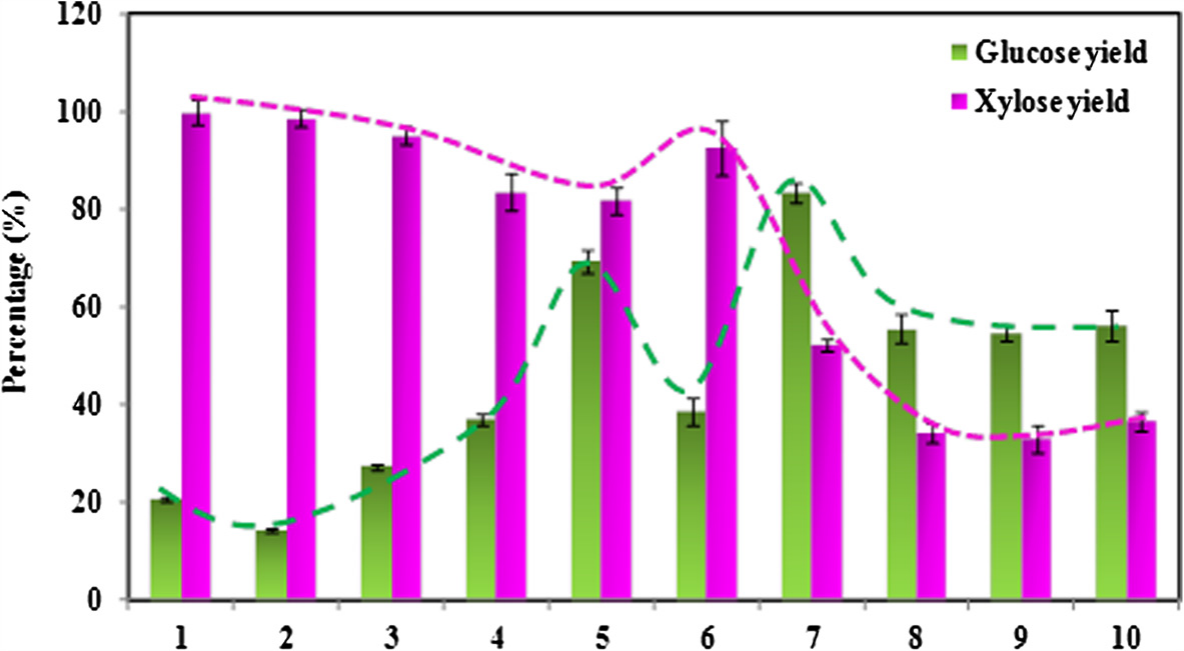
**Glucose and xylose yield after acidolysis of switchgrass in [C**_**4**_**mim]Cl.** The number of the x-axis label corresponds to the run numbers in Tables 2 and 3.

It was previously reported that rapid “flash” heating (higher temperature and shorter time) using certain ILs to pretreat biomass is more efficient with regards to the carbohydrate yield and removal of lignin [25]. Previous results from our group have typically used temperatures between 120–160°C and time intervals of 1–3 h [26]. In order to define the best set of conditions for pretreatment and acidolysis, switchgrass was processed using different pretreatment combinations of temperature, time and water addition protocols, and the results are compared in Table 2 and Figure 3. After pretreatment at 160°C for 1.5 h and 5% solids loading, particles were not observed and the mixture was a homogenous dark solution. Thus, the pretreatment time under 160°C was not carried out longer than 1.5 h. The sugar yields obtained after pretreatment and hydrolysis were calculated using Eq. 4:

(4) $$\mathrm{Yield}\;\%=\frac{C_{\sup}\times TV_{\sup}}{W_{\mathrm{sg}}\times C_{\mathrm{sug}}\times f}\times 100\%$$

where, *TV*_*sup*_ is the total volume of the supernatant, *W*_*sg*_ is the weight of the switchgrass pretreated by IL, *C*_*sug*_ is the percentage of glucan/xylan contained in the switchgrass, and *f* is the factor to convert glucan/xylan to glucose/xylose (1.11 for glucan and 1.136 for xylan). The results show that the final dilution is not necessary since the glucose yield is improved by 35% with 37% more water in the system and xylose yields are similar. With increased temperature and decreased pretreatment time the glucose yield increased from 20.7% to 69.4%, while xylose recovery decreased from 99.8% to 81.9%, possibly due to enhanced xylose degradation at higher temperatures. Compared to the reported data, these glucose yields are lower under the 105°C/6 h pretreatment condition [27]. We attribute these differences to the different types of biomass used (corn stover *vs.* switchgrass), types of IL ([C_2_mim]Cl vs. C_4_mim]Cl), and the scale of the biomass loading (26.7 mg g corn stover + 502 mg IL *vs.* 0.5 g SG + 10 g IL), all of which are essential factors that affect the final yields.

**Table 2:** **Glucose and xylose yields after acidolysis of biomass in [C**_**4**_**mim]Cl using different pretreatment conditions and water addition methods**^**a**^

**Run no.**	**Pretreatment conditions**	**H**_**2**_**O addition method**	**Solid residue (wt%)**	**Glucose yield (%)**	**Xylose yield (%)**
1	105°C 6 h	Aliquot	34.9	20.7 ± 0.4	99.8 ± 2.6
2^b^	105°C 6 h	Aliquot	34.8	24.2 ± 0.3	98.6 ± 1.8
3^c^	105°C 6 h	Aliquot	34.8	27.4 ± 0.7	95.1 ± 2.0
4	160°C 1 h	Aliquot	13.7	37.1 ± 1.2	83.4 ± 3.8
5	160°C 1.5 h	Aliquot	6.7	69.4 ± 2.5	81. 9 ± 2.8
6	160°C 1.5 h	Pumped @ 10 min	13.8	38.6 ± 2.7	92.6 ± 5.5
7	160°C 1.5 h	Pumped @ 15 min	10.2	83.3 ± 1.9	52.1 ± 1.2

^a^ For all runs, 0.5 g biomass was mixed with 10 g [C_4_mim]Cl; ^b^ 7.5 mL water was added at the end of the acidolysis; ^c^ 15 mL water was added at the end of the acidolysis.

In previous reports [27], water was added at defined time intervals (0 min, 10 min, 20 min, 30 min and 60 min) during acidolysis to achieve higher sugar yields. As this may not be a practical approach to water addition, we evaluated the impact of pumping water into the system using a syringe pump, and the results are compared from runs 5 to 7 Table 2. Runs 6 and 7 indicate that the time interval when the water was pumped into the system has a significant impact on the observed sugar yield. For example, in run 6, water was pumped in 10 min after the initial addition of water and acid; the glucose yield was observed to decrease significantly (Run 5 69.4% *vs*. Run 6 38.6%) although they were pretreated under the same conditions: 160°C for 1.5 h. When the reaction interval was changed to 15 min with 20% water addition performed at 10 min, the glucose yield improved from 69.4% to 83.3%. We hypothesize that this result can be attributed to the kinetics of the glucose hydrolysis based on observations made in previous reports [27], and the optimized water addition are plotted in Additional file 1: Figure S2. Figure 3 shows clearly that the maximum glucose yields occur at the expense of reduced xylose yields, which is expected, as xylan is easier to hydrolyze compared to glucan. At more severe process conditions, more glucan can be broken down but this also results in spontaneous xylose degradation.

As concentrated sugar hydrolysates are favored for downstream fermentation, we explored the impact of higher switchgrass solid loading levels, and the results are shown in Table 3. Although more solid residue (absolute mass) remains after the pretreatment-acidolysis process and the glucose/xylose yields decrease (83.3% *vs.* 54.4% for glucose and 52.1% *vs.* 32.9% for xylose), the final glucose/xylose concentration in the system increased to 15.5 g/L for glucose and 6.9 g/L for xylose. This may indicate that in order to obtain high sugar concentrations at high yields, a multi-cycle pretreatment process using this approach should be considered.

**Table 3:** **Glucose and xylose yields after acidolysis of biomass in [C**_**4**_**mim]Cl using different solids loadings**^**a**^

**Run No.**	**Solid loading**^**b **^**(w/w)**	**Solid residue (wt%)**	**Glucose yield (%)**	**Xylose yield (%)**	**Final [Glc] (g/L)**	**Final [Xyl] (g/L )**
7	5	10.2	83.3 ± 1.9	52.1 ± 1.2	8.0 ± 0.2	3.4 ± 0.1
8	7.5	9.7	55.5 ± 3.0	34.1 ± 2.0	8.3 ± 0.4	3.5 ± 0.2
9	10	9.4	54.4 ± 1.6	32.9 ± 2.8	10.2 ± 0.3	6.7 ± 0.4
10	15	8.0	56.2 ± 3.1	36.7 ± 1.9	15.5 ± 0.8	6.9 ± 0.4

^a^ water was pumped into the system 15 min after pretreatment.

^b^ solid loading is the ratio of the mass of SG to mass of IL.

### Sugar extraction after acidolysis of biomass

**Figure 4:**
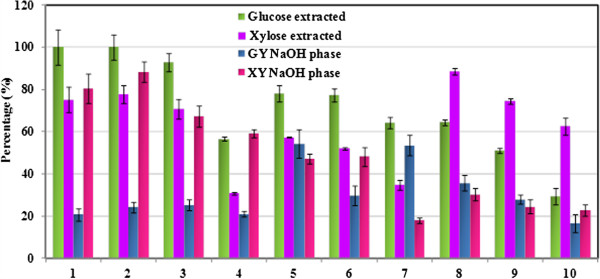
**Percentage of glucose and xylose partitioned to the alkaline phase (first two columns) and final sugar yields (GY: glucose yield, XY: xylose yield) in the alkali phase (last two columns, calculated according to Eq. 5).** The number of the x-axis label corresponds to the run numbers in Tables 2 and 3.

After the acidolysis step, the supernatant was separated from the solid biomass residue by centrifugation. 1 mL supernatant was loaded in eppendorf tubes and the calculated amount of concentrated NaOH was added into the tubes. After vigorous mixing the solution was centrifuged and clear evidence of phase separation was observed (Figure 1b). Sugar extraction was calculated based on the percentage of sugars in the alkaline phase to the sugars present in the supernatant before phase separation (Eq. 1), and the results are presented in Figure 4 and Table 4. Overall, more glucose partitioned to the alkaline phase compared to xylose as observed by comparing the first two columns in Figure 4 (Runs 1–7). With higher solid loadings, more xylose partitioned to the lower phase indicating the effect of biomass components such as lignin or carbohydrate oligomers on sugar partitioning. With higher glucose yields (e.g. Run 7: 83.3% vs. Run 1: 20.7%) from the acid hydrolysis, less sugar partitioned to the lower phase (Run 7: 64.1% vs. Run 1: 98.5%). After extraction, less than 2% of the sugars remain in the [C_4_mim]Cl phase. The final sugar yields in the last two columns of Table 4 (GY_NaOH_, XY_NaOH_) represent how much sugar was recovered in the alkaline phase, calculated using Eq. 5:

(5) $$GY_{\mathrm{NaOH}}\mathrm{or}\;XY_{\mathrm{NaOH}}=Y_{\mathrm{sug}}\times E_{\mathrm{sug}}\times 100\%$$

where *Y*_*sug*_ is the sugar yield calculated according to Eq. 4 and *E*_*sug*_ is the extraction percentage calculated based on Eq. 1. Overall, up to 54% of the glucose or 88% of xylose in original switchgrass can be released and then extracted to the alkaline phase (different pretreatment conditions are required depending on whether C5 or C6 is the focus). With more glucose recovered, less xylose can be obtained and a limited amount (< 0.06 g/L) of sugars were detected in the [C_4_mim]Cl phase, ca. 50% of the sugars were lost after the process under the optimized conditions (Run 5 with final sugar yields in alkaline phase: 54.23% for glucose; 47.01% for xylose). It was reported that 82% glucose yield and less than 18.5% xylose was obtained in the hydrolysate after pretreatment of switchgrass (3% w/w biomass loading) in 1-ethyl-3-methylimidazolium acetate ([C_2_mim]OAc) at 160°C for 3 h followed by 72 h enzymatic saccharification [28]. Although the glucose yield from the acidolysis-extraction process is lower than that reported using the more conventional IL pretreatment approach, the significantly higher xylose recovery, elimination of the extensive washing steps normally required to remove IL residue in pretreated biomass, and elimination of the costly hydrolytic enzymes suggest that this process may offer compelling economic advantages. The partition coefficients for glucose fall into the same range as those obtained with the sugar standards (2.96–3.39 vs. 2.91). The partition coefficients for xylose are less than those for glucose (2.58–3.28). At higher biomass loadings (Runs 8–10), the partition coefficients for both glucose and xylose are observed to decrease, and the variation of logP between runs indicates again that other biomass components present in the solution may interfere with partitioning.

**Table 4:** Partition of the sugars after phase separation

**Run no.**	**[Glc]**_**IL **_**(g/L)**^**a**^	**[Glc]**_**NaOH **_**(g/L)**	**Log P**_**glc**_	**[Xyl]**_**IL **_**(g/L)**^**a**^	**[Xyl]**_**NaOH **_**(g/L)**	**Log P**_**xyl**_	**GY**_**NaOH **_**(%)**^**b**^	**XY**_**NaOH **_**(%)**^**b**^
1	0.004	8.55 ± 0.65	3.33	ND	18.58 ± 1.50	-	22.64	80.33
2	0.002	2.31 ± 0.13	3.06	ND	7.47 ± 0.41	-	15.60	88.26
3	0.001	2.46 ± 0.11	3.39	0.002	3.62 ± 0.24	3.26	25.26	67.19
4	0.007	8.84 ± 0.13	3.10	0.003	5.71 ± 0.10	3.28	20.98	59.05
5	0.016	17.84 ± 0.27	3.05	0.005	8.24 ± 0.05	3.22	54.23	47.01
6	0.008	10.07 ± 0.17	3.10	0.005	8.10 ± 0.17	3.21	29.81	48.15
7	0.018	24.73 ± 1.39	3.14	0.006	7.00 ± 0.65	3.07	53.42	18.07
8	0.020	26.56 ± 0.28	3.12	0.040	15.20 ± 0.49	2.58	35.72	30.23
9	0.023	28.06 ± 0.26	3.09	0.041	26.91 ± 0.42	2.82	27.82	24.46
10	0.036	32.94 ± 2.86	2.96	0.057	30.86 ± 3.19	2.73	16.57	22.91

Note: [Glc]_IL_ or [Xyl]_IL_ is glucose or xylose concentration in the IL phase; [Glc]_NaOH_ or [Xyl]_NaOH_ is glucose or xylose concentration in NaOH phase; ND: not detected. ^a^ The standard deviation is within 20% of the measurement. The deviation is due to the low concentration of the sugars in IL phase and detection limit of the instrument. ^b^GY_NaOH_ is the final glucose yield in NaOH phase (percentage of the glucose in original biomass); XY_NaOH_ is the final xylose yield in NaOH phase (percentage of the xylose in original biomass).

Sugars can also be lost due to spontaneous degradation to other small molecules during acidolysis and/or sugar extraction. For example, glucose and xylose can be dehydrated to furans and other degradation products under acidic conditions [29]. In order to determine the extent to which this occurred during acidolysis, 5-(hydroxymethyl)furfural (HMF) and furfural were quantified in the supernatant as well as the upper and lower phases, and the results are listed in Table 5. The results show that only up to 5% of the glucose (equivalent) was converted to HMF after acidolysis, but more xylose (5–11%) was dehydrated to furfural. Neither furfural nor HMF can be detected by liquid chromatography in the two phases after the addition of concentrated NaOH, but further work is needed to verify these results as it is expected that these degradation products would partition with the sugars.

**Table 5:** Quantification of the HMF and furfural in the system

**Run no.**	**% Glu. to HMF**	**% Xyl. to furfural**
1	2.16 ± 0.00	6.07 ± 0.01
2	1.79 ± 0.01	6.05 ± 0.02
3	2.05 ± 0.01	5.63 ± 0.02
4	2.95 ± 0.01	5.88 ± 0.01
5	4.79 ± 0.06	8.52 ± 0.02
6	3.25 ± 0.03	9.01 ± 0.02
7	5.14 ± 0.02	10.93 ± 0.00
8	3.66 ± 0.02	4.66 ± 0.04
9	3.20 ± 0.00	6.23 ± 0.01
10	4.53 ± 0.00	9.35 ± 0.07

The [C_4_mim]Cl content was determined in the alkaline phase and the results are listed in Table 6. The percentage of the [C_4_mim]Cl present in the lower phase was found to be dependent on the pretreatment conditions. At higher temperature and shorter pretreatment time (Runs 4–10), less than 1% of the [C_4_mim]Cl in the supernatant is observed to partition to the alkaline phase. Comparatively, 5–9% of the [C_4_mim]Cl partitioned to the lower phase for runs 1–3 at lower temperature and longer pretreatment time. The highest [C_4_mim]Cl content was found in the alkaline phase with 15 mL water dilution at the end of the acidolysis. NMR analysis (Additional file 1: Figure S3) shows that the [C_4_mim]Cl present in the upper phase is the same as the signal obtained from the original [C_4_mim]Cl, while no signal can be detected in the lower (alkaline) phase with NMR.

**Table 6:** Quantification of the IL in the lower alkaline rich phase

**Run**	**Dilution (mL)**	**[C**_**4**_**mim]Cl in alkali phase (mM)**	**% of IL to the alkaline phase**
1	0	784.5	5.8
2	1.5	302.6	5.9
3	3	317.0	9.4
4	0	75.2	0.6
5	0	63.5	0.4
6	0	70.6	0.5
7	0	39.9	0.3
8	0	97.2	0.6
9	0	90.3	0.5
10	0	86.1	0.4

### Mass balance and characterization of the solid residue

**Figure 5:**
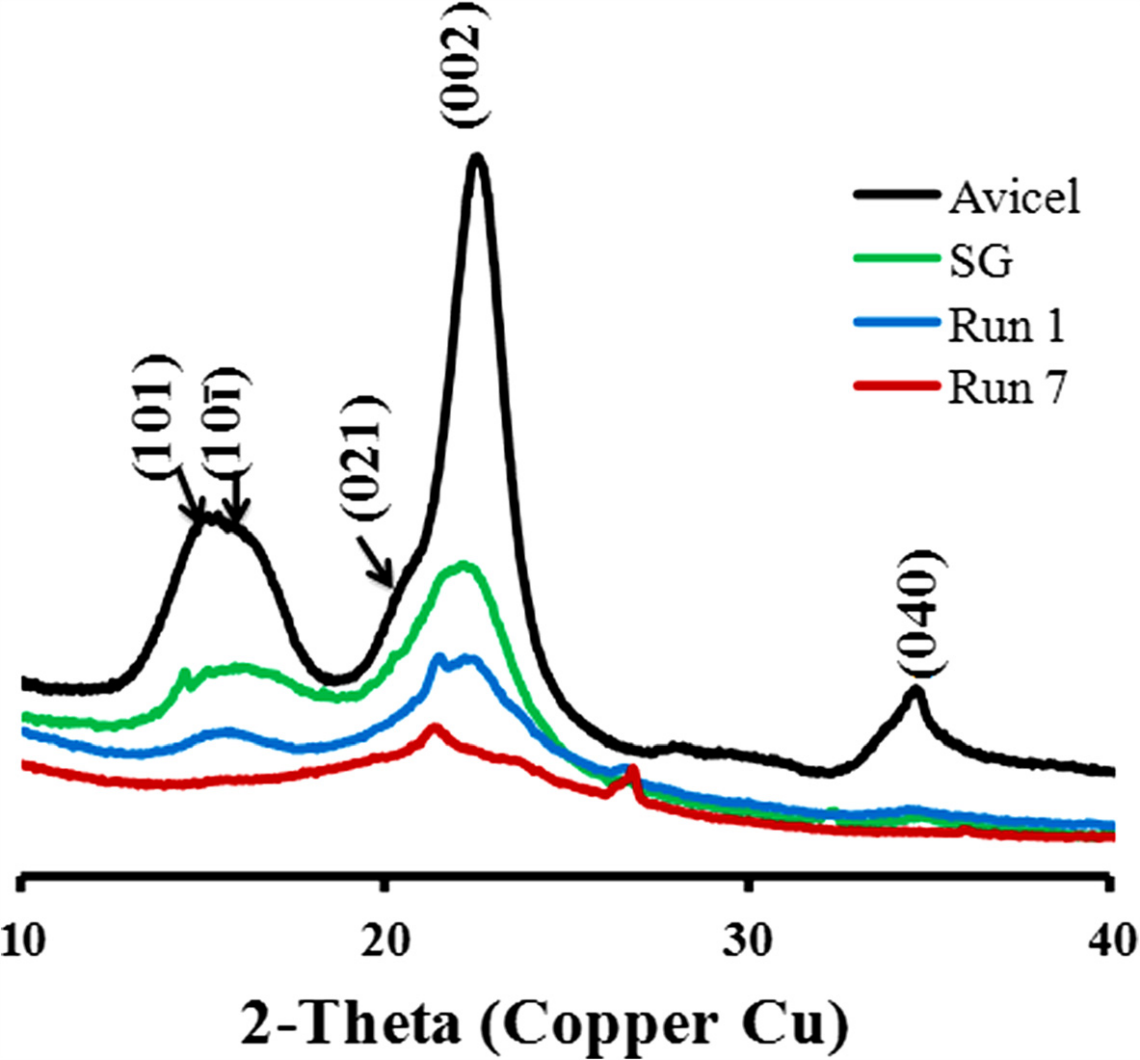
**Diffractograms of the biomass before and after the process.** Red, Avicel, Green, switchgrass, Blue: solid residue from run 1, Purple: solid residue from run 7. CrI of Avicel: 0.74, SG: 0.38, Run 1: 0.29, Run 5: 0.08.

After the acidolysis process, a small quantity of solid was obtained. This solid residue is expected to be the most recalcitrant part of the plant cell wall, and different analytical techniques were used to characterize the material. X-ray diffraction (XRD) was used to determine the proportions of crystalline (highly ordered) and disordered components (amorphous cellulose, hemicelluloses and lignin) present in biomass samples, and to monitor the structural changes upon [C_4_mim]Cl treatment and acidolysis. Commercial Avicel was used as a cellulose standard to validate the results. In general, the solid residue recovered after pretreatment and acidolysis has a significant reduction in the degree of crystallinity present as compared to the untreated switchgrass (Figure 5). The XRD patterns are observed to be dependent on the pretreatment conditions. At the lower pretreatment temperature (Run 1, T = 105°C), solid residue after pretreatment and acidolysis displays a crystal structure very similar to that of the original biomass (cellulose I). In addition to cellulose I peaks (15–16° for 101 and 10ī, 22° for 002), a shoulder around 21.5° is also observed, suggesting at least partial conversion to cellulose II. Overall, the calculated CrI shows a decrease from 0.38 to 0.29 with pretreatment at 105°C for 6 h.

In contrast, the biomass pretreatment at higher temperature results in disappearance of the broad peak at ca. 15–16º, which represents a combination of the 101 and 10ī planes of cellulose I. The material is highly amorphous with a minor crystalline component (CrI = 0.08). The broad peak around 21.4° may be assigned to the 002 cellulose II lattice plane. This indicates that the solvent IL has penetrated inside the solid part and disrupted the crystal structure of cellulose during the higher temperature pretreatment. This may explain why higher temperature/shorter time pretreatment is more efficient in solubilizing the biomass, thus resulting in more sugar production. Another possible explanation for the observed structural change is that the relative ratio of the three major biomass components is altered as a result of the pretreatment.

**Figure 6:**
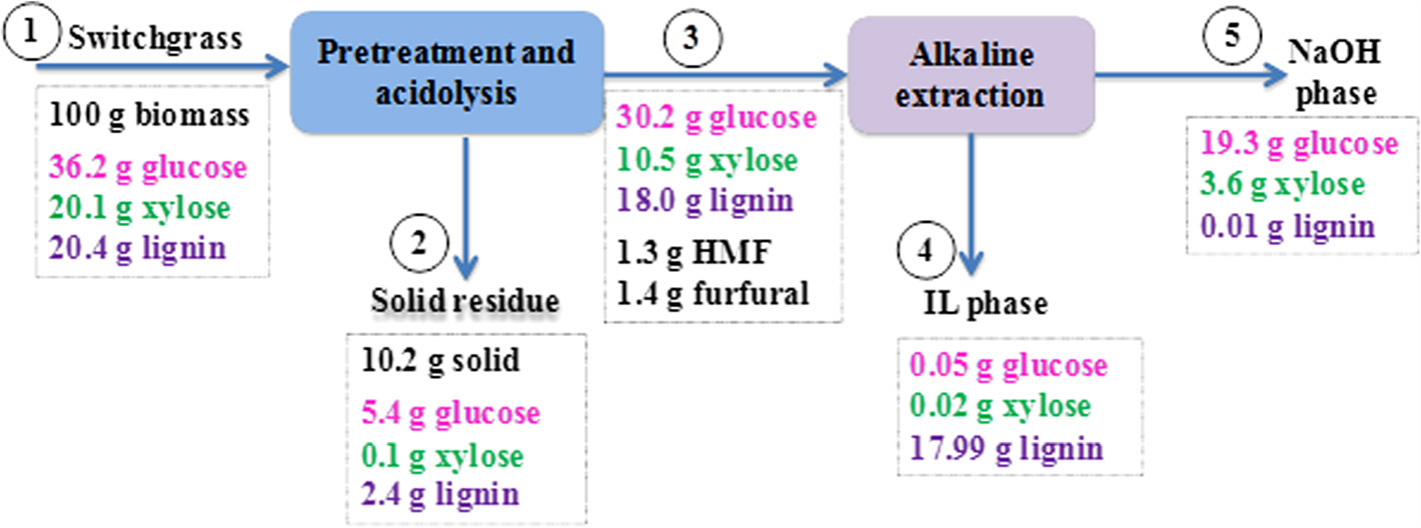
**Representative mass balance of lignocellulose as defined by the process conditions used in Run 7.** Lignin/sugars in the solid samples (stream 1 and 2) were quantified using the standard method [33]; lignin in NaOH phase (stream 5) was quantified gravimetrically by adjusting the pH of the solution to pH = 2–3 using 4 N HCl; lignin in stream 3 and 4 was calculated by subtraction; sugars in the liquid stream (3–5) were quantified using HPAEC.

Glucan, xylan and lignin (acid insoluble) contents of the solid residue were quantified and the results are listed in Additional file 1: Table S1. After the pretreatment and acidolysis process, the glucan contents in the solid residue are generally higher as compared to their relative abundance in the original biomass. Xylan contents are all under 5%, indicating most of xylan has been solubilized/hydrolyzed. Lignin content in the solid residue is generally close to the original switchgrass for higher temperature pretreatment (Run 7–10). A mass balance for Run 7 is shown in Figure 6, and it should be noted that less xylose is observed in the NaOH phase, and that a significant amount of the lignin remains in the IL phase and would need to be removed in order to recycle the IL.

**Figure 7:**
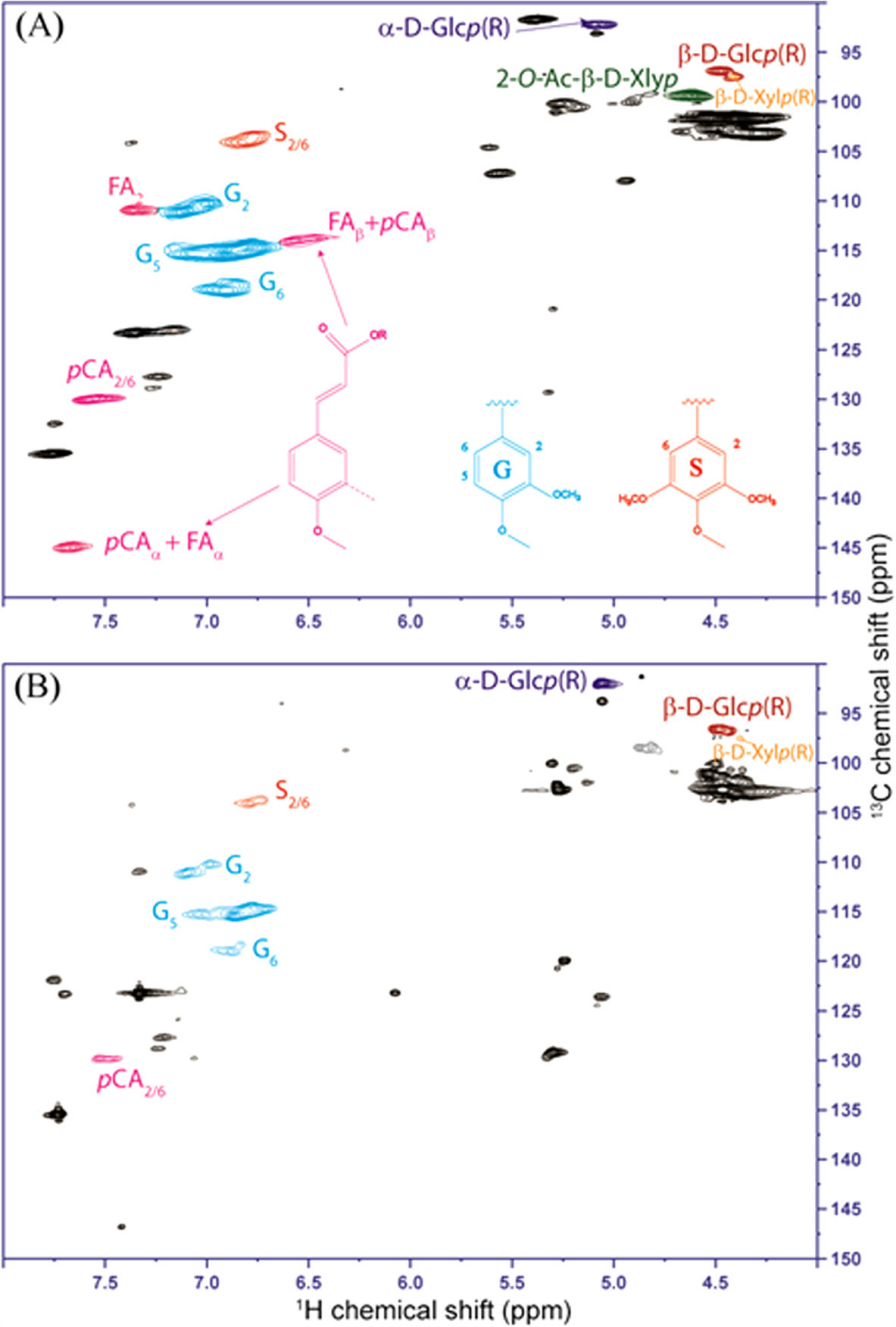
2D 13C-1H correlation (HSQC) spectra from gel samples in DMSO-d6/pyridine-d5 (4:1) for the sampes: original switchgrass (A) and solid residue from run 7 (B).

The solid residue was analyzed with 2D NMR to further define the solid residue and the spectra are shown in Figure 7. It has been shown that a mixture of perdeuterated DMSO/pyridine (4:1, v/v) is a better solvent compared to DMSO-*d6* only with regards to sample handling as well as the resolution and intensities of NMR spectra [30], and thus a mixture of perdeuterated DMSO/pyridine (4:1, v/v) was chosen as the solvent. The aromatic region (4.0–5.5/102–150 ppm) of the 2D HSQC spectra provides information on the p-hydroxyphenyl:guaiacyl:syringyl (H:G:S) distributions in the lignin. According to the spectra in Additional file 1: Figure S4, the switchgrass lignin is dominated by G lignin with less amount of H and S units which is consistent with previous results presented in the literature [31]. The correlation of S2/6 (6.78/104.02) and H2/6 (7.24/127.8; 7.27/128.93) are very weak can only be seen at a low contour level. The C/H correlations from the G aromatic rings (G2, G5 and G6) are well resolved for both samples, except that G5 (6.88/115.55) is overlapping with ferulate (FA) and *p*-coumarate (*p*CA): FAβ + PCAβ (6.58/113.83). Ferulates and *p*-coumarates, attached primarily to arabinoxylans, are readily observed in grass samples [30,32]. The peak at 7.39/111.03 is assigned to FA2, and FA6 appears at 7.16/123.20 which is overlapping with the solvent peak (pyridine). After the process the signals decreased and FA6 can be only observed with low contour level. The *p*CA2/6 correlations are well resolved at 7.58/130.09 and *p*CA3/5 position is not resolved from G5 units. FAα and FAβ correlations coincide with *p*CAα and *p*CAβ respectively at positions 7.67/145.08 and 6.58/113.83 pm. Integrals from well resolved 2,6-positions of each type of lignin can be used to calculate the H:G:S ratio. The integration results for the two samples are shown in Additional file 1: Figure S4. In general, it was observed that all types of lignin signals have been weakened after the pretreatment and hydrolysis process. The S/G ratio decreased from 0.32 to 0.21 after the processing. The anomeric regions (4–5.5/90–105 ppm) indicate that xylan has been mostly removed with the disappearance of the peaks at 4.60/99.47 and 4.39/101.77 ppm which corresponds to xylan acetate (2-Ο-Ac-β-D-Xyl*p*) and xylan [(1 → 4)-α-D-Glc*p*]. The intensities of the peaks for cellulose reducing ends [Glc(R), 5.08/92.27 & 4.46/96.98] have been greatly enhanced in the solid residue, indicating a lower degree of polymerization (DP) after the process. These results indicate that the solid residue has experienced significant compositional and chemical changes after the acidolysis and extraction process. Most of the xylan has been removed, whereas lignin and cellulose has been left with modified structures. 2D NMR shows detailed bonding structures, however, the dissolution of the samples in the solvent mixture was not complete, and may not be a representative fraction of the whole solid residue.

The data presented in Table 7 and Additional file 1: Figure S5 show the molecular distribution of lignin in the switchgrass lignin extracted using the enzymatic mild acidolysis lignin (EMAL) as a control, supernatant after acidolysis, as well as the IL phase and NaOH phase after phase separation. The data have been presented as large molecular weight material (t < 15.5 min elution time, corresponding to 46 k < u by polystyrene calibration) and small molecular weight material (t > 15.5 min elution time corresponding to 46 k > u). It is observed that after pretreatment and acidolysis the largest component present in the EMAL (7–9 min elution time) is no longer present in the supernatant. Moreover, a significant reduction in larger molecular weight material (< 15.5 min elution time) and an increase in the smaller molecular weight materials (> 15.5 min) were observed. This result indicates that lignin has undergone significant depolymerization during this pretreatment/acidolysis process at the conditions used. The component of large molecular weight material is 70.1% before pretreatment but is reduced to 46.2% after acidolysis. Furthermore, after phase separation, lignin fractionation is observed between the two phases; only 4.7% of larger molecular weight material is present in the IL phase, with the remaining larger molecular weight material migrating to the NaOH phase. The partition of different molecular mass lignin after phase separation gives rise to possible fractionation method for lignin.

**Table 7:** **Lignin molecular weight distribution (calculated from SEC data in Additional file**1**: Figure S5)**

**Elution time (min)**	**t < 15**	**t > 15**
Molecular mass (u)*	**46 k < u**	**46 k > u**
EMAL	70.1%	29.9%
Before phase separation	46.2%	53.8%
IL phase	4.7%	95.3%
NaOH phase	69.8%	30.2%

*Based on polystyrene calibration.

## Conclusions

It has been shown that certain concentrations of NaOH can phase separate with chloride based ILs ([C_4_mim]Cl and [C_2_mim]Cl) forming upper phase, IL rich and lower phase, alkaline rich. Both glucose and xylose prefer to partition to the alkaline rich phase. By combining this system with the acidolysis of biomass in IL, sugar monomers can be easily extracted from the aqueous ILs. The sugar yields depend on both the pretreatment conditions and alkali concentrations. Pretreatment under higher temperature results in improved glucose yields but compromises xylose yields. The optimized NaOH concentration for both phase separation and sugar extraction is 15%. Maximum yields of 54% glucose and 88% xylose can be recovered in the alkaline phase with pretreatment condition of 160°C for 1.5 h and 105°C for 6 h respectively followed by acidolysis. Improved sugar yields could be achieved by further optimizing the amount of acid and water used in acidolysis step and the alkali salts used for sugar extraction. Molecular dynamics simulations can be used to predict sugar partitioning in the system, but the sugar partition coefficients are found to be affected by the presence of biomass.

Consolidated pretreatment and hydrolysis using ILs and acid catalysts offers a promising route to the production of fermentable sugars without the need for enzymes. The use of alkali salts to form ABS to recover the sugars and recycle the IL may provide a scalable and economical process. The advantages of the process are: 1) sugars can be released *in situ* and extracted by alkaline solution with relatively high yields, and without the need for any enzymes; 2) the formation of an aqueous biphasic system enables facile recovery of the sugars and IL recovery at the same time; 3) significantly reduced volume of water (< 50 wt% of total mixture) is used as compared to more traditional IL based pretreatment process. Future research should be focused on recovery of the residual biomass in the IL rich phase, test the IL recycling efficiency, and desalting of the alkaline rich phase to make it compatible with downstream fermentation microbes.

## Methods

### Biomass pretreatment and acidolysis of dissolved biomass

The pretreatment and acidolysis process flow is shown in Additional file 1: Figure S6. Biomass solutions were prepared by combining different amounts (0.5 g, 0.75 g, 1 g, and 1.5 g) of switchgrass with 10 g [C_4_mim]Cl in an 80 mL glass bottle. The mixtures were heated and stirred in an oil bath at different conditions. All experiments were conducted in duplicates. Solutions were then placed into another oil bath which was already equilibrated at the acidolysis temperature of 105°C and acidolysis started after 15 min equilibration.

Acidolysis was performed following a procedure described previously [27]. In summary, 4 M HCl was added to the biomass-[C_4_mim]Cl solution (t = 0) at concentrations of 100 mg HCl per g biomass and with DI water added to give a H_2_O concentration of 5% (w/w) of the total weight. More water was added at different time intervals (10 min, 20 min, 30 min, and 60 min) to result in targeted water concentrations of 20, 25, 33 and 43%. Continuous water addition using a syringe pump was also attempted to compare the effect on sugar yields. Water was pumped into the mixture starting from either 10 or 15 min at the rate of 157.2 or 121.1 uL/min for 50 or 45 min. Acidolysis was continued for a total of 2.5 h and stopped by taking the bottle out of the oil bath with/without addition of extra amount of water (0, 7.5, or 15 mL). The mixture was transferred into centrifuge tubes and centrifuged at high speed (10,000 rpm) to separate the solid residue from the aqueous solutions. The solid residue was washed with 5× 40 mL of water, and after the final wash the sample was lyophilized for two days for further analysis.

### Extraction of sugars using alkaline solution

#### Extraction of sugar standards

33 mg glucose and 21 mg xylose were used to simulate the sugar outputs obtained from 0.1 g biomass. These sugars were dissolved in an IL-H_2_O mixture (2 g IL + 1.5 g H_2_O) in a 15 mL centrifuge tube. 70 μL of 4 M HCl was added to the mixture and mixed in an incubator at 30°C and 1400 rpm for 30 min. 1 mL of the mixture solution was placed in to a 2 mL eppendorf tube and different amounts (ca. 130 or 200 uL) of concentrated NaOH (50% w/w) were added to give the final NaOH concentration either 15 or 20 wt% (considering the water in the system). The mixture was agitated in a thermomixer at RT and 1400 rpm for 0.5 h and then centrifuged at high speed (14,000 rpm) to phase separate. The upper IL phase and lower NaOH phase were separated with a pipette and the sugar content was quantified. The volume of the upper and lower phase was calculated by measuring the mass and density of both phases.

#### Extraction of acidolysis sugars

The procedure is similar to the extraction of sugar standard except that only 15 wt% NaOH (final concentration, based on the water in the system) was used based on the results from the sugar standards. The total volume of the supernatant was calculated based on the total mass and density of the supernatant after separation of the solid residue.

### Analysis and characterization methods

All aqueous solutions were analyzed for sugars using High Performance Anion Exchange Chromatography with Pulsed Amperometric Detection (HPAEC-PAD) on a Dionex ICS 3000 equipped with a Dionex CarboPac PA-20 analytical column (3 × 150 mm), according to procedures described previously [8,33]. Elution was initiated with 89% (v/v) water and 11% (v/v) 1 M NaOH for the first 13.5 min, with 10 μL injection volume and 0.4 mL/min for the flow rate. A 5 min gradient was applied and elute concentration was then switched to 55% (v/v) water and 45% (v/v) 100 mM NaOH for 30 min. Sugar standards fucose, arabinose, rhamnose, galactose, glucose, xylose, fructose, and cellubiose obtained from Sigma-Aldrich were used as the external standards for HPAEC, and prepared at levels of 6.25 to 100 μM before use.

Furfural and HMF was analyzed using an Agilent 1200 High Pressure Liquid Chromatography (HPLC) instrument equipped with Aminex HPX-87 H column and a UV detector ( λ = 280 nm). Eluent containing 4 mM H_2_SO_4_ was used and the flow rate was 0.6 mL/min. Standard calibration curves were made by using 6 different known concentrations of furfural/HMF (125–1000 uM) from Sigma-Aldrich. Ionic liquid was quantified using reversed phase liquid chromatography using an HPLC equipped with Eclipse Plus C8 column and Evaporative Light Scattering Detector (ELSD, evaporator temperature = 45°C, nebulizer temperature = 30°C; gas flow = 1.2). All analyses were performed at 0.5 mL/min flow rate. The injection volume was 5 μL and the column temperature was 30°C.

The chemical composition of the biomass before and after pretreatment was tracked using an acidolysis protocol that followed the NREL Laboratory Analytical Protocols (LAP) LAP-002 and LAP-005 scaled down to the volumes of the samples [34]. In short, 0.2 g biomass and 2 mL 72% H_2_SO_4_ was incubated at 30°C with shaking rate of 300 rpm for 1 h. The solution was diluted down to 4% H_2_SO_4_ and autoclaved for 1 h at 121°C. The reaction was quenched by placing the samples into an ice bath and then filtered. Carbohydrate concentrations were determined using HPAEC and the acid insoluble lignin was quantified gravimetrically.

XRD data were collected with a PANalytical Empyrean X-ray diffractometer equipped with a PIXcel^3D^ detector and operated at 45 kV and 40 kA using Cu *Kα* radiation (λ = 1.5418 Å). The patterns were collected in the 2θ range of 5 to 55°, the step size was 0.026°, and the exposure time was 300 seconds. A reflection-transmission spinner was used as a sample holder and the spinning rate was set at 8 rpm throughout the experiment. The crystallinity index (CrI) was determined from the crystalline and amorphous peak areas by a curve fitting procedure of the measured diffraction patterns using the software package HighScore Plus^®^ according to Eq. 6:

(6) $$\mathrm{CrI}=\frac{\sum A_{\mathrm{cr}.}}{\sum A_{\mathrm{cr}.}+\sum A_{\mathrm{am}.}}$$

NMR samples were ball milled using a Retsch PM 100 planetary ball mill at 600 rpm with a stainless steel grinding jar (50 mL) containing zirconium dioxide balls (10 mm × 10). The samples were milled for 6 h with 5 min grinding intervals with 5 min breaks. 25 mg of these milled biomass samples were mixed with 500 uL pre-mixed DMSO-*d6*/pyridine-*d5* (4:1 v/v) directly in NMR tubes according to the report by Ralph *et al*[30]. The tubes were placed into an ultrasonic bath with the temperature at 50°C and for 8 h to swell/dissolve the biomass. Heteronuclear single quantum coherence (HSQC) spectra were recorded at 310 K using a Bruker Avance-600 MHz equipped with a cryo-probe (hsqcetgpsisp.2, ns = 128, ds = 16, d1 = 0.5 s, td = 1 k, number of increments = 512). DMSO was used as an internal reference. Topspin was used for processing and analysis of the data.

Size exclusion chromatography (SEC) was employed to assess changes in lignin mass distribution. An EMAL lignin of switchgrass was employed as a control [35]. EMAL has shown to be more representative of the total lignin present in biomass compared to the lignin extracted using other protocols such as milled wood lignin (MWL) or cellulolytic enzyme lignin (CEL) [36,37]. Lignin solutions were prepared in analytical grade N-methyl-2-pyrrolidinone (NMP) and dimethylsulfoxide (DMSO) (1:1, v/v) with sonication for 3 hours at 40°C. Polydispersity of dissolved lignin was determined using analytical techniques SEC UV-A as previously described [38]. An Agilent 1200 series binary LC system (G1312B) equipped with a DAD (G1315D) was used. Separation was achieved with a Mixed-D column (5 mm particle size, 300 mm × 7.5 mm i.d., linear molecular weight range of 200 to 400,000 u, Polymer Laboratories) at 80°C using a mobile phase of NMP at a flow rate of 0.5 ml min^-1^. Absorbance of material eluting from the column was detected at λ = 300 nm (UV-A). Molecular mass estimates were determined after calibration of the system with polystyrene standards.

### Molecular dynamics simulation of glucose in IL-water-NaOH system

Data from molecular dynamics (MD) simulations were used to calculate the partition coefficients of the glucose in the water-IL-sugar-NaOH system to explain the partitioning of glucose to the upper (IL rich) phase and the lower (NaOH rich) phase. Four systems (Table 8) were constructed using PACKMOL [39]. To simplify the systems for MD in order to obtain a realistic approximation of the interactions of glucose with each phase, we designed the MD simulation systems to match the concentration of each component (either [C_2_mim]Cl in IL phase or NaOH in basic phase) to correspond to the experimental data. The appropriate amount of glucose was added to both the upper and lower phases, and MD simulations were carried out in each of the two phases. Two reference systems of the two phases without glucose were also studied using MD simulations. MD simulations were implemented in AMBER [40], using an all-atom force field based on the generalized AMBER force field (GAFF) [41] for [C_2_mim] cation [42], the standard Cl^-^ anion force field for chloride ions, and the carbohydrate force field, GLYCAM [43], for glucose. The cutoff radius of non-bonded interactions was set to 8 Å. The Particle Mesh Ewald summation method [44] was used to calculate the electrostatic potential with periodic boundary conditions. The initial configurations were energy minimized using the steepest descent algorithm for 1000 steps to remove any unexpected coordinate collisions. The systems were then heated for 200 ps in the NVT ensemble, during which the temperature was increased gradually to the target temperature (300 K), followed by a further 500 ps of equilibration dynamics in the NPT ensemble using a Nose-Hoover constant pressure (P = 1 bar) control algorithm. Finally, 10 ns production runs were carried out in the NPT ensemble. The time step for production runs was 2 fs and the SHAKE algorithm was employed to constrain bonds and angles involving hydrogen atoms. The last 8 ns of each trajectory were used for analysis. The simulation box sizes, types of molecule and their numbers, and related parameters for the various systems are given in Table 8.

**Table 8:** The component of simulated systems

	**Number of NaOH**	**Number of water**	**Number of [C**_**2**_**mim]Cl**	**Number of glucose**
NaOH phase with sugar	90	300	0	8
NaOH phase	90	300	0	0
IL Phase with sugar	0	200	32	8
IL phase	0	200	32	0

## Competing interests

The authors declare that they have no competing interests.

## Authors’ contribution

NS carried out the biomass pretreatment, acidolysis, sugar extraction and sugar analysis work, performed the NMR analysis, and drafted the manuscript. HL and KLS carried out the MD simulation for the system and drafted the simulation related parts of the manuscript. NS and AG performed the SEC analysis of the lignin and drafted the SEC related parts of the manuscript. VS performed the PXRD analysis and drafted the PXRD related parts of the manuscript. MS and AB participated the compositional analysis. KT contributed to development of phase separation process. BMH contributed to the IL content measurement. SS, BAS and BMH contributed to the original experimental design and general guidance of the paper. All authors suggested modifications to the draft and approved the final manuscript.

## Supplementary Material

Additional file 1: Table S1 Compositions of the major components in the solid residue. **Figure S1** Chromatograms of the upper IL-rich phase (red, 5× dilution) and lower salt-rich phase (blue, 3000× dilution) for standard sugars. **Figure S2** The addition of water at different time intervals. More water is needed at 10 min to achieve high sugar yields. **Figure S3a **^13^C NMR of the original IL, upper (IL rich) phase, and lower (salt rich rich) phase. **Figure S3b **^1^H NMR of the original IL, upper (IL rich) phase, and lower (salt rich) phase. **Figure S4** Lignin integration results based on 2D NMR spectra. Blue: raw switchgrass, Red: solid residue after processing. **Figure S5** SEC chromatogram of EMAL lignin from switchgrass (green), supernatant after acidolysis (blue) and IL phase (red). **Figure S6** Process of biomass pretreatment, acid hydrolysis and sugar extraction using alkaline solutions. Click here for file
