# Supplementary material for: Production and extraction of sugars from switchgrass hydrolyzed in ionic liquids
Source: Biotechnol Biofuels. 2013 Mar 20;6:39. doi: 10.1186/1754-6834-6-39 (PMC3621597; doi:10.1186/1754-6834-6-39)
Supplement: Additional file 1: Table S1 — Compositions of the major components in the solid residue. Figure S1 Chromatograms of the upper IL-rich phase (red, 5× dilution) and lower salt-rich phase (blue, 3000× dilution) for standard sugars. Figure S2 The addition of water at different time intervals. More water is needed at 10 min to achieve high sugar yields. Figure S3a 13C NMR of the original IL, upper (IL rich) phase, and lower (salt rich rich) phase. Figure S3b 1H NMR of the original IL, upper (IL rich) phase, and lower (salt rich) phase. Figure S4 Lignin integration results based on 2D NMR spectra. Blue: raw switchgrass, Red: solid residue after processing. Figure S5 SEC chromatogram of EMAL lignin from switchgrass (green), supernatant after acidolysis (blue) and IL phase (red). Figure S6 Process of biomass pretreatment, acid hydrolysis and sugar extraction using alkaline solutions. [file 1754-6834-6-39-S1.pdf]

Supplementary Information

Table S1 Compositions of the major components in the solid residue

Run number	Glucan	Xylan	AIL
**1**	80.6	4.8	13.4
**7**	53.3	1.1	23.9
**8**	70.2	1.2	19.0
**9**	80.0	1.7	16.2
**10**	71.8	1.5	19.4
**SG**	36.2	20.1	20.4

Standard deviation is within 10% of the measured value.
SG: switchgrass
AIL: acid insoluble lignin

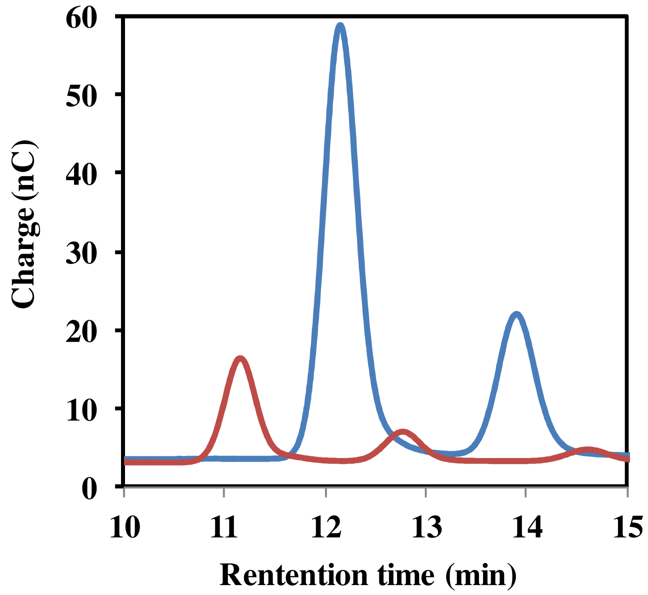

Figure S1. Chromatograms of the upper IL-rich phase (red, 5x dilution) and lower salt-rich phase (blue, 3000x dilution) for standard sugars.

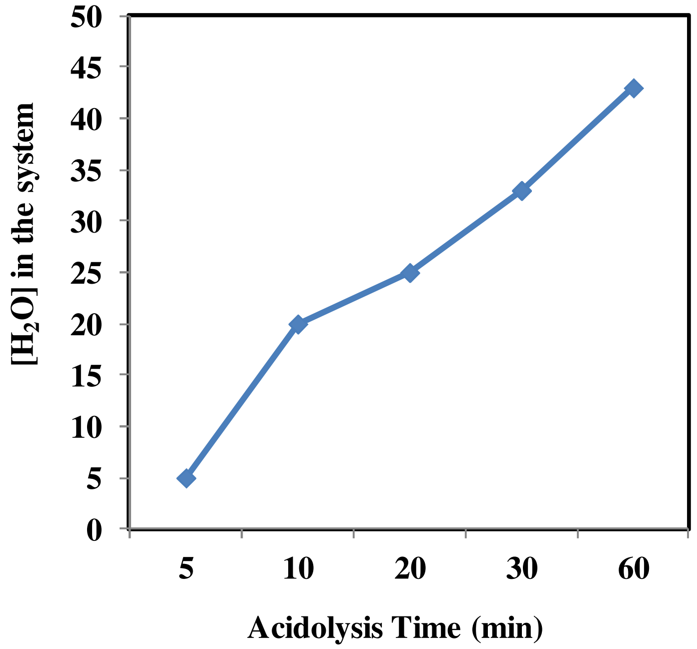

Figure S2. The addition of water at different time intervals. More water is needed at 10 min to achieve high sugar yields.

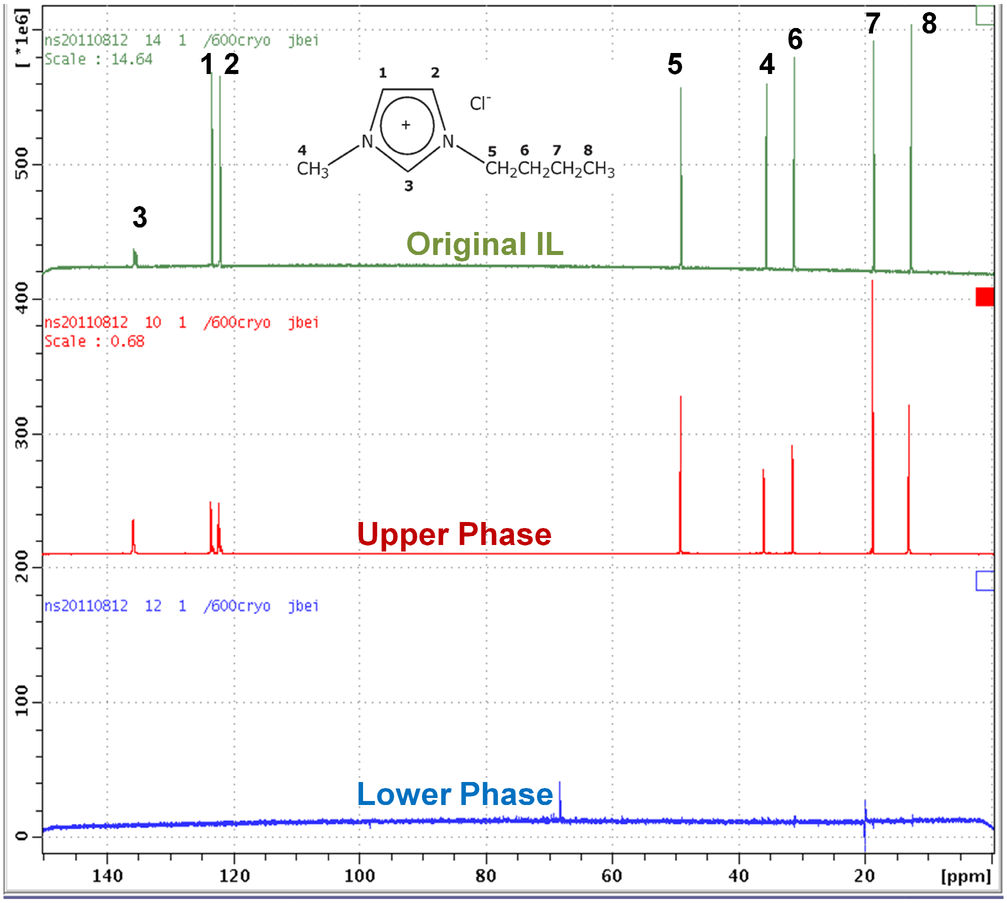

Figure S3a. ^{13}C NMR of the original IL, upper (IL rich) phase, and lower (salt rich) phase.

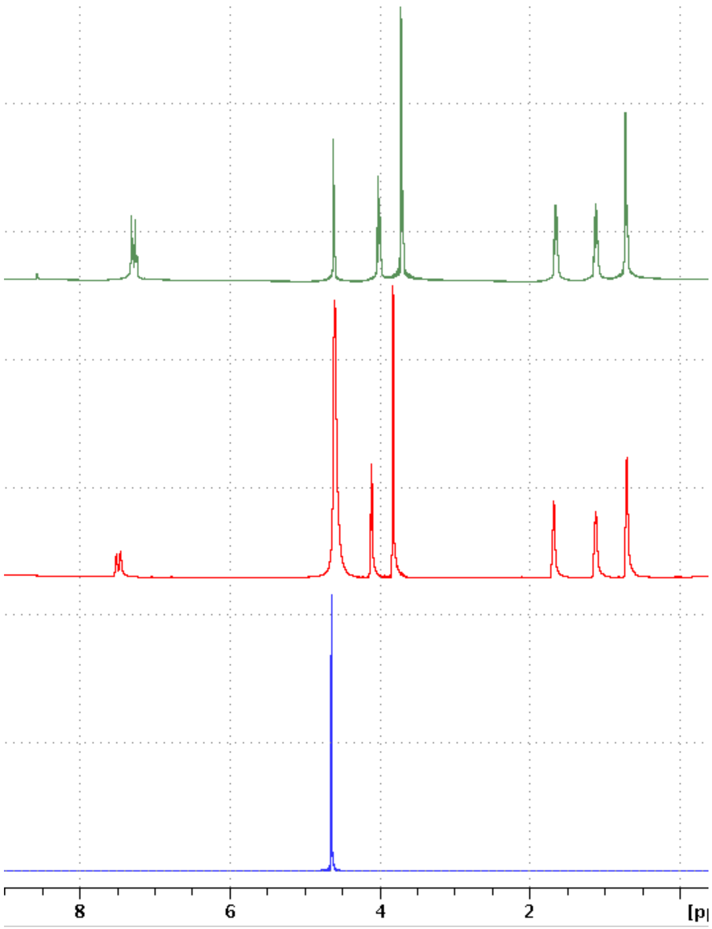

Figure S3b. ^{1}H NMR of the original IL, upper (IL rich) phase, and lower (salt rich) phase.

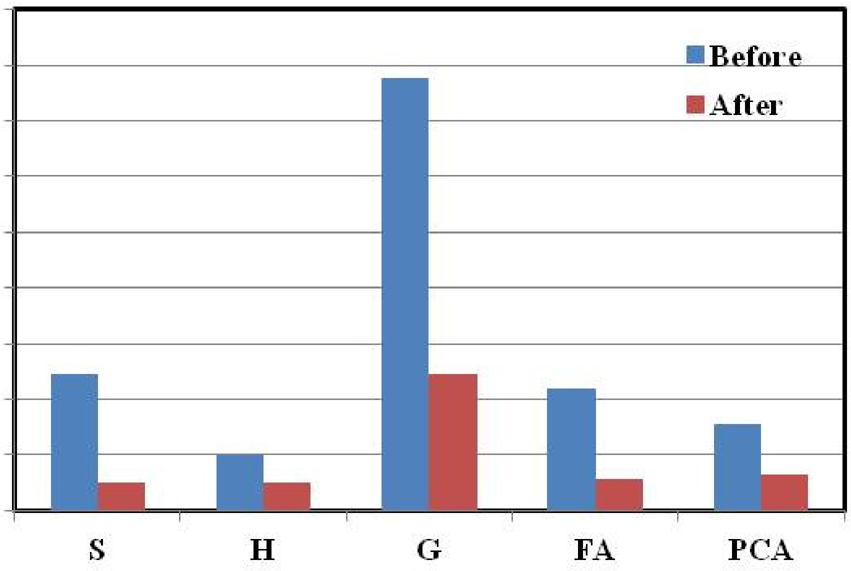

Figure S4. Lignin integration results based on 2D NMR spectra. Blue: raw switchgrass, Red: solid residue after processing.

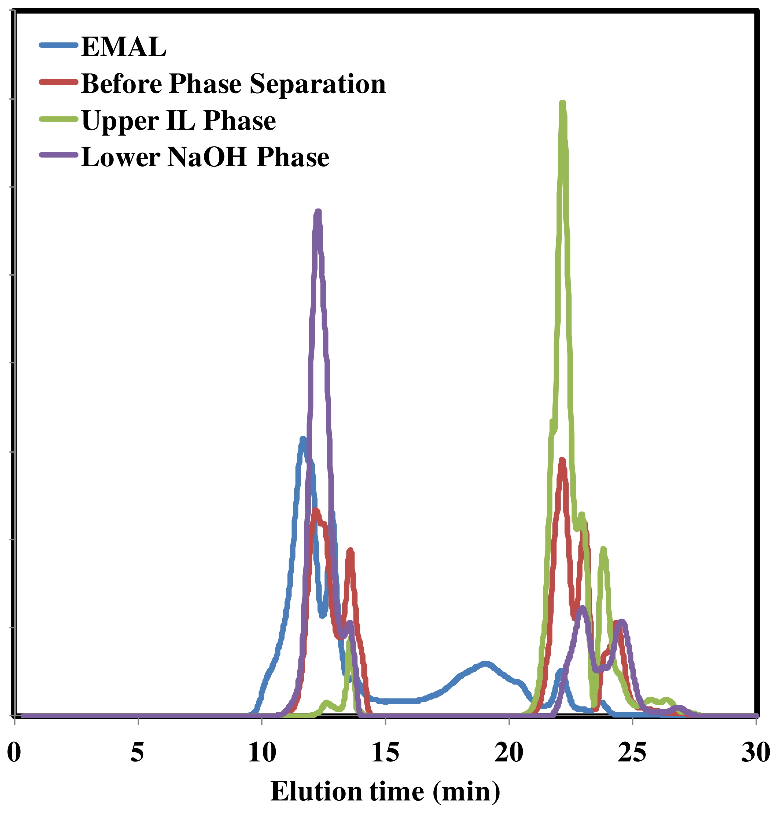

Figure S5 SEC chromatogram of EMAL lignin from switchgrass (green), supernatant after acidolysis (blue) and IL phase (red)

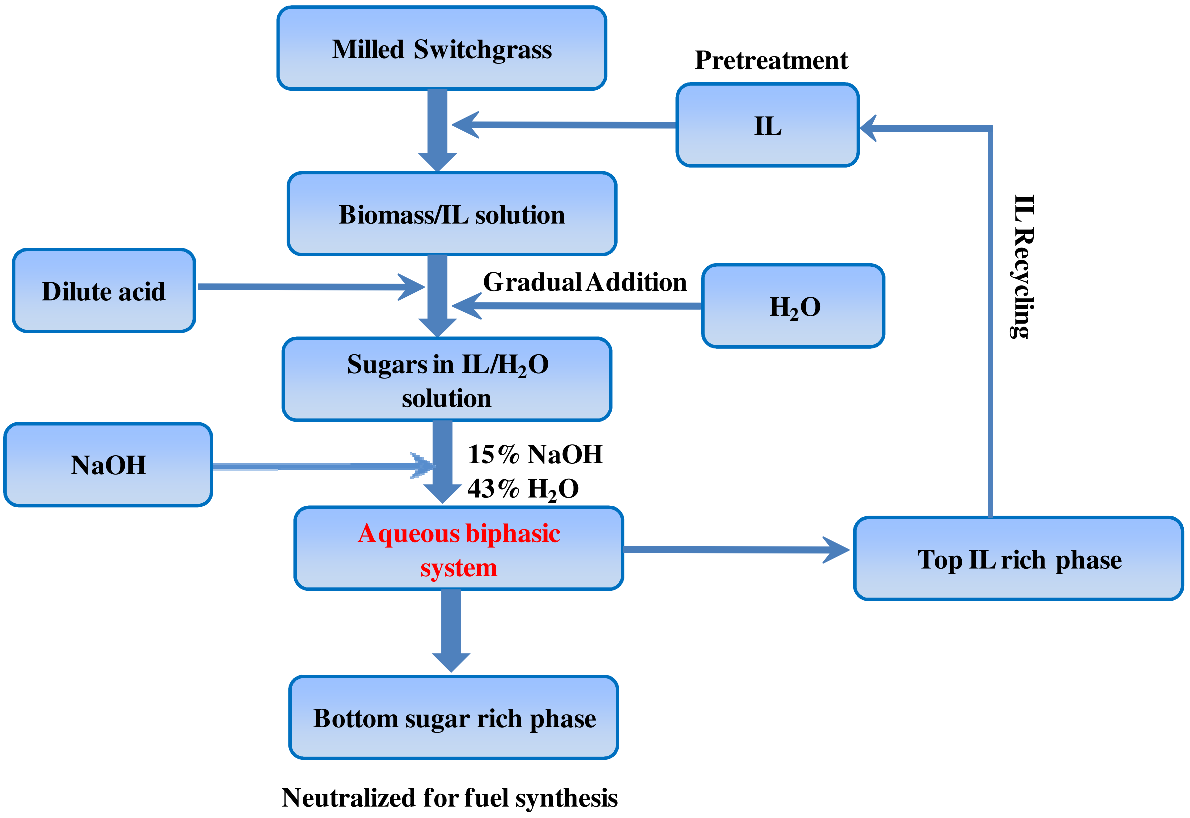

Figure S6. Process of biomass pretreatment, acid hydrolysis and sugar extraction using alkaline solutions.
